# A Modified *in vitro* Clot Lysis Assay Predicts Outcomes in Non-traumatic Intracerebral Hemorrhage Stroke Patients—The IRONHEART Study

**DOI:** 10.3389/fneur.2021.613441

**Published:** 2021-04-20

**Authors:** Rita Orbán-Kálmándi, Tamás Árokszállási, István Fekete, Klára Fekete, Máté Héja, Judit Tóth, Ferenc Sarkady, László Csiba, Zsuzsa Bagoly

**Affiliations:** ^1^Division of Clinical Laboratory Sciences, Department of Laboratory Medicine, Faculty of Medicine, Kálmán Laki Doctoral School University of Debrecen, Debrecen, Hungary; ^2^Department of Neurology, Faculty of Medicine, University of Debrecen, Debrecen, Hungary; ^3^Department of Radiology, Faculty of Medicine, University of Debrecen, Debrecen, Hungary; ^4^Hungarian Academy of Sciences (MTA-DE) Cerebrovascular and Neurodegenerative Research Group, University of Debrecen, Debrecen, Hungary

**Keywords:** hemorrhagic stroke, clot lysis, outcome, intracerebral hemorrhage, neutrophil extracellular traps

## Abstract

**Background:** Non-traumatic intracerebral hemorrhage (ICH) accounts for 10–15% of all strokes and results in a higher rate of mortality as compared to ischemic strokes. In the IRONHEART study, we aimed to find out whether a modified *in vitro* clot lysis assay method, that includes the effect of neutrophil extracellular traps (NETs) might predict ICH outcomes.

**Patients and Methods:** In this prospective, observational study, 89 consecutive non-traumatic ICH patients were enrolled. Exclusion criteria included aneurysm rupture, cancer, liver- or kidney failure or hemorrhagic diathesis. On admission, detailed clinical and laboratory investigations were performed. ICH volume was estimated based on CT performed on admission, day 14 and 90. A conventional *in vitro* clot lysis assay (CLA) and a modified CLA (mCLA) including cell-free-DNA and histones were performed from stored platelet-free plasma taken on admission. Clot formation and lysis in case of both assays were defined using the following variables calculated from the turbidimetric curves: maximum absorbance, time to maximum absorbance, clot lysis times (CLT) and area under the curve (CLA AUC). Long-term ICH outcomes were defined 90 days post-event by the modified Rankin Scale (mRS). All patients or relatives provided written informed consent.

**Results:** Patients with more severe stroke (NIHSS>10) presented significantly shorter clot lysis times of the mCLA in the presence of DNA and histone as compared to patients with milder stroke [10%CLT: NIHSS 0–10: median 31.5 (IQR: 21.0–40.0) min vs. NIHSS>10: 24 (18–31.0) min, *p* = 0.032]. Shorter clot lysis times of the mCLA showed significant association with non-survival by day 14 and with unfavorable long-term outcomes [mRS 0–1: 36.0 (22.5.0–51.0) min; mRS 2–5: 23.5 (18.0–36.0) min and mRS 6: 22.5 (18.0–30.5) min, *p* = 0.027]. Estimated ICH volume showed significant negative correlation with mCLA parameters, including 10%CLT (*r* = −0.3050, *p* = 0.009). ROC analysis proved good diagnostic performance of mCLA for predicting poor long-term outcomes [AUC: 0.73 (0.57–0.89)]. In a Kaplan-Meier survival analysis, those patients who presented with an mCLA 10%CLT result of >38.5 min on admission showed significantly better survival as compared to those with shorter clot lysis results (*p*=0.010).

**Conclusion:** Parameters of mCLA correlate with ICH bleeding volume and might be useful to predict ICH outcomes.

## Introduction

Stroke is a leading cause of death and disability in all developed countries. Non-traumatic intracerebral hemorrhage (ICH) constitutes ~10–15% of acute strokes and has a much higher risk of morbidity and mortality than ischemic strokes or subarachnoid hemorrhage (1, 2). Despite advances in acute stroke care, pharmacological treatment in ICH is still limited, and it remains the most devastating cerebral vascular disease with a mortality of up to 30–50% (1). Coagulation disorders and impaired hemostasis have been shown to increase the risk of ICH (3, 4). However, studies on associations between hemostasis or fibrinolysis abnormalities and the outcome of ICH remains limited. Besides a handful of studies indicating that increased admission D-dimer levels predict mortality (5–7), the impact of the fibrinolytic system on the outcome of acute non-traumatic ICH has not been fully investigated.

The clot lysis assay (CLA) is a global assay of the fibrinolytic system reflecting the overall plasma fibrinolytic potential (8). This test has been used to study clot formation and fibrinolysis in a wide spectrum of pathologies, including acute ischemic stroke (8–13). Although the CLA is feasible and has a potential for clinical use to examine fibrin clot properties and fibrinolysis, the assay is not free of analytical challenges and protocols used in different laboratories may vary significantly. Moreover, the assay is performed using plasma and therefore potential cellular contributors of hemostasis and fibrinolysis are not incorporated in the test. Recent data identified neutrophil extracellular trap (NET) components, released from activated neutrophils as important modulators of fibrinolysis (14–16). NETs are networks of DNA and histones, decorated with granular neutrophil proteins (e.g., elastase), originally described as a first line defense mechanism of the innate immune system (17). It has been shown that NETs intercalate to fibrin and create a dense network that is resistant to fibrinolysis (14–16). NETs have been implicated as important constituents of venous and arterial thrombi, and the prothrombotic and antifibrinolytic effects of NETs have been in the focus of wide range of research in recent years (14, 18–25).

The aim of this study was to evaluate whether a modified CLA (mCLA) incorporating the effect of NETs might predict outcomes in a cohort of patients with non-traumatic, spontaneous ICH.

## Materials and Methods

### Patients

In this prospective observational study, consecutive patients with non-traumatic intracerebral hemorrhage stroke (ICH) were enrolled in a single stroke center (Department of Neurology, University of Debrecen, Hungary). Patient enrollment started in June 2017 and finished in September 2020. Inclusion criteria were: patients over 18 years of age with acute non-traumatic intracerebral hemorrhage, verified with non-contrast computerized tomography (NCCT) scan. Exclusion criteria included the presence of cerebral aneurysm, AV malformation, epidural hemorrhage, subdural hemorrhage, malignancy, severe hepatic- and renal insufficiency, hemorrhagic diathesis and SARS-CoV-2 infection at hospital admission or during follow-up. The presence of ICH was diagnosed by complex neurological examination based on clinical symptoms, brain imaging using NCCT scan. Follow-up NCCT scans were performed 14 days and 3 months after the event. CT images were analyzed simultaneously by 3 independent investigators and a comprehensive list of radiographic features and estimated ICH volume was recorded (26). For each patient, the time of symptom onset, demographic and clinical characteristics (age, sex, BMI, previous medications, history of cerebrovascular and cardiovascular diseases, cerebrovascular risk factors including smoking) were registered on admission. Stroke severity was determined by the National Institutes of Health Stroke Scale (NIHSS) on admission and on day 7 (27). Risk stratification of each patient was performed using the ICH score (based on GCS score, age, infratentorial origin, intraventricular hemorrhage and ICH volume) (28). Patients were followed and long-term functional outcomes were determined at 3 months after the stroke event using the modified Rankin Scale (mRS) (29). As from March 2020, all patients were investigated about potential acquiration and symptoms of SARS-CoV-2 infection on admission and during follow-up. In case of a suspected infection, the diagnosis was confirmed by a routine method of reverse transcriptase polymerase chain reaction testing of RNA extracted from nasopharyngeal/oropharyngeal swabs.

The following outcomes were investigated: 1/Mortality by day 14 and day 90. 2/Long-term outcome at 90 days post-event: mRS 0–1 was defined as favorable long-term outcome (30).

### Informed Consent

The study design was in accordance with the guiding principles of the Declaration of Helsinki and was approved by the Institutional Ethics Committee of the University of Debrecen and the Ethics Committee of the National Medical Research Council. All patients or their relatives provided written informed consent.

### Blood Sampling and Laboratory Measurements

Peripheral venous blood samples were taken from all patients on admission. Routine laboratory tests (ions, glucose level, renal and liver function tests, high-sensitivity C-reactive protein measurement, complete blood count) were carried out immediately by standard laboratory methods (Roche Diagnostics, Mannheim, Germany and Sysmex Europe GmbH, Hamburg, Germany). For the examination of hemostasis tests, blood samples were collected to vacutainer tubes containing 0.109 M sodium citrate (Becton Dickinson, Franklin Lane, NJ) and were processed immediately (centrifugation twice at 1,500 g, room temperature, 15 min). Screening tests of coagulation (prothrombin time, activated partial thromboplastin time, and thrombin time) were performed immediately on a BCS coagulometer using routine methods (Siemens Healthcare Diagnostic Products, Marburg, Germany). For the execution of *in vitro* clot lysis assays (CLA and mCLA) and other specific hemostasis tests, aliquots of citrated plasma were labeled with a unique code and stored at −80°C until analysis. Specific hemostasis tests including CLA and mCLA were performed from stored plasma aliquots by investigators blinded to patient identification and clinical data. Fibrinogen levels were measured according to the method of Clauss on a BCS coagulometer (Siemens Healthcare Diagnostic Products, Marburg, Germany). Plasminogen and α2-plasmin inhibitor (α2-PI) activities were measured by commercially available methods on a BCS coagulometer. Plasma levels of FXIII activity were determined by ammonia release assay using a commercially available reagent kit (REA-chrom FXIII kit, Reanalker, Budapest, Hungary).

### *In vitro* CLA and mCLA Measurements

Recombinant t-PA-driven lysis of tissue factor-induced plasma clots was studied in 96-well microtiter plates by monitoring changes in turbidity. Assay conditions in our study were based on previously described methods, with some modifications (8, 13, 31–33). Two assay conditions were used, and plasma samples were run in quadruplicates in both assay conditions. All concentrations provided refer to final concentrations in the 100 μL final well volume. Plasma samples were thawed in a water bath at 37°C. In the first assay condition (CLA), citrated plasma was mixed with 1,000-fold diluted human tissue factor (Innovin, Siemens, Marburg, Germany) and 100 ng/ml rt-PA (Alteplase, Boehringer Ingelheim, Ingelheim, Germany) in HEPES buffer (10 mM HEPES, 150 mM NaCl, 0.05% Tween 20, pH:7.4). In order to mimick the effect of NETs, in the second assay condition (mCLA) 150 μg/ml pure and cell-free DNA (cfDNA) (calf thymus DNA, Sigma-Aldrich, Darmstadt, Germany) and 50 μg/ml calf thymus histone (TIII S, Calbiochem, La Jolla, CA, USA) were also added to the sample solutions. Optimal concentrations of cfDNA and histones were tested in preliminary experiments based on literature where the combined effect of histones (50 μg/ml) and various concentrations of cfDNA (50–250 μg/ml) were studied on fibrinolysis kinetics in purified experimental conditions (16). Dilution of plasma samples with buffer was 1.2-fold in case of both assay conditions. Clot formation in both conditions was initiated by automated sample pipetting of HEPES buffer, containing 21 mM CaCl_2_, to each sample well. Optical density was measured at 340 nm, 37°C every minute for 300 min in a TECAN Infinite m200 microplate reader (TECAN Trading AG, Männedorf, Switzerland). Curves were analyzed using the Shiny app software tool (34). The following parameters were calculated from the turbidimetric curves in case of both assay conditions: maximum absorbance, time to maximum absorbance, various points of clot lysis time (CLT): 10% clot lysis time (10%CLT), 50%CLT, 90%CLT and area under the curve (CLA AUC). Clot lysis times were defined as the time from the 10, 50, or 90% point, from clear to maximum turbidity, to the 10, 50, or 90% point, respectively, in the transition from maximum turbidity to the final baseline turbidity (10%CLT, 50%CLT, and 90%CLT parameters, respectively). Analytical precision of both assay conditions was evaluated according to the guidelines of Clinical and Laboratory Standards Institutes (CLSI document EP05-A3) (35, 36). Precision was tested using healthy control plasmas, each run in quadruplicate, for 20 days. Coefficients of variation (CVs) of the within-run and total (within-laboratory) precision assessments were 8.6 and 8.9%, respectively. Precision results were essentially similar in both assay conditions. Representative CLA and mCLA curves and reference parameters of healthy individuals as compared to patients are provided as Supplementary Figure 1 and Supplementary Table 1.

### Statistical Analysis

Statistical analysis was performed using the Statistical Package for Social Sciences (SPSS, Version 26.0, Chicago, IL), and GraphPad Prism 8.0 (GraphPad Prism Inc., La Jolla, CA). Normality of data was studied using the Shapiro-Wilk-test. Student's *t*-test or Mann–Whitney *U*-test was performed for independent two-group analyses. In case of paired data, paired *t*-test or Wilcoxon signed-rank-test was applied. ANOVA with Bonferroni *post-hoc*-test or Kruskal–Wallis analysis with Dunn's *post-hoc*-test was used for multiple comparisons. Spearman's correlation coefficient was used to determine the strength of correlation between continuous variables. Differences between categorical variables were assessed by χ^2^-test or by Fisher's exact where appropriate. Receiver operating characteristic (ROC) curves were built by plotting sensitivity vs. 1-specificity and calculating the area under the curve (AUC). Optimal threshold values were calculated based on Youden's J statistics. Test characteristics of sensitivity, specificity, positive predictive value (PPV), and negative predictive value (NPV) were calculated using contingency tables and χ^2^-test or Fisher's exact at statistically optimal threshold values. The Kaplan-Meier method was applied to plot survival vs. non-survival of patients, based on the calculated optimal test parameter cut-off. Survival curves were compared using the log-rank test. Binary backward logistic regression models were used to determine independent predictors of mortality and long-term functional outcome. Adjustments of the models were based on the results of preliminary statistical analyses of baseline characteristics between groups (Student's *t*-test or Mann–Whitney *U*-test, χ^2^-test or Fisher's exact), literature data, and methodological principles (dichotomized variables when possible). Results of the logistic regression analysis were expressed as odds ratio (OR) and 95% confidence interval (CI). A *p*-value of <0.05 was considered statistically significant.

## Results

In the IRONHEART study, 89 patients with non-traumatic, spontaneous ICH were enrolled. One patient was excluded from the study due to SARS-CoV-2 infection on admission. One patient acquired SARS-CoV-2 infection on day 25 after the event, thus long-term follow-up results were excluded in this case. The assumed cause of ICH was hypertension in all patients, as based on the exclusion criteria, other causes, including cerebral aneurysm, AV malformation, malignancy, severe liver insufficiency, hemorrhagic diathesis, amyloidosis or vasculitis were excluded. Baseline characteristics of patients, imaging data and outcomes are shown in Table 1. The mean age of the cohort was 68 (± 11.6) years, 64% of patients were men. Median NIHSS on admission was 14 (IQR: 8–20), median ICH score was 1 (IQR: 1–3). The most frequent cerebrovascular risk factor was hypertension (96.6%). Screening tests of coagulation and fibrinogen levels did not indicate a hemorrhagic defect in any of the patients. The median volume of hemorrhage was 20.0 (IQR: 3.7–48.0) cm^3^ on admission and 46 (51.7%) of patients had intraventricular hemorrhage extension. Mortality was 29.0% within the first 14 days after event and 43.8% by day 90.

**Table 1:** Baseline characteristics of enrolled patients, imaging data and outcomes.

**Number of patients, *n***	**89**
Age, y, mean ± SD	68 (±11.6)
Male sex, *n* (%)	57 (64.0)
Stroke severity on admission, NIHSS, median (IQR)	14 (8–20)
Stroke severity on discharge, NIHSS, median (IQR)	19 (8–43)
ICH score, median (IQR)	1 (1–3)
Glasgow coma scale, median (IQR)	13 (9–14)
**Cerebrovascular risk factors**, ***n*** **(%)**	
Arterial hypertension	86 (96.6)
Atrial fibrillation	11 (12.4)
Diabetes mellitus	39 (43.8)
Hyperlipidemia	48 (53.9)
Active smoker	15 (16.9)
BMI, kg/m^2^, median (IQR)	27.0 (24.1–31.9)
**Laboratory measurements on admission, median (IQR)**	
INR	0.97 (0.93–1.05)
APTT, s	27.7 (25.4–31.2)
WBC, G/L	8.6 (6.7–11.5)
Platelet count, G/L	226 (170–265)
Serum glucose, mmol/L	7.6 (6.0–10.4)
hsCRP, mg/L	2.7 (1.2–6.5)
Creatinine, μmol/L	69.0 (61.0–84.5)
Fibrinogen, g/L	3.8 (3.1–4.4)
Plasminogen activity (%)	110 (100–122)
α2- plasmin inhibitor activity (%)	107 (98–113)
Factor XIII activity (%)	166 (139–176)
**Imaging data**, ***n*** **(%)**	
**Presence of hydrocephalus on admission**	
No	59 (66.3)
External hydrocephalus	2 (2.2)
Internal hydrocephalus	21 (23.6)
Both	7 (7.9)
**Hemisphere localization of ICH on admission**	
Left hemisphere	44 (49.4)
Right hemisphere	41 (46.1)
Bilateral hemisphere	4 (4.5)
**Presence of intraventricular hemorrhage on admission**	
No	43 (48.3)
Subarachnoideal	11 (12.4)
Lateral ventricule	8 (9.0)
III. ventricule	1 (1.1)
IV. ventricule	1 (1.1)
Combined	25 (28.1)
**Infratentorial origin**	
Yes	4 (4.5)
No	85 (95.5)
**Estimated volume of hemorrhage, cm**^**3**^**, median (IQR)**	
On admission	20.0 (3.7–48.0)
Day 14	10.0 (2.8–27.0)
Day 90	0 (0.0–2.4)
**Outcomes**, ***n*** **(%)**	
Mortality by day 14	26 (29.0)
**Long-term outcome (mRS, day 90)**	
Favorable (mRS 0–1)	15 (16.9)
Unfavorable (mRS 2–5)	32 (36.0)
Death (mRS 6)	39 (43.8)
Undetermined	3 (3.3)

*Data are means ± SD or medians (interquartile ranges). APTT, activated partial thromboplastin time; BMI, body mass index; hsCRP, high sensitivity C-reactive protein measurement; ICH, intracerebral hemorrhage, INR, international normalized ratio; IQR, interquartile range; i.v., intravenous; mRS, modified Rankin Scale; NIHSS, National Institutes of Health Stroke Scale; WBC, white blood cell*.

### Clot Lysis Results

**Figure 1:**
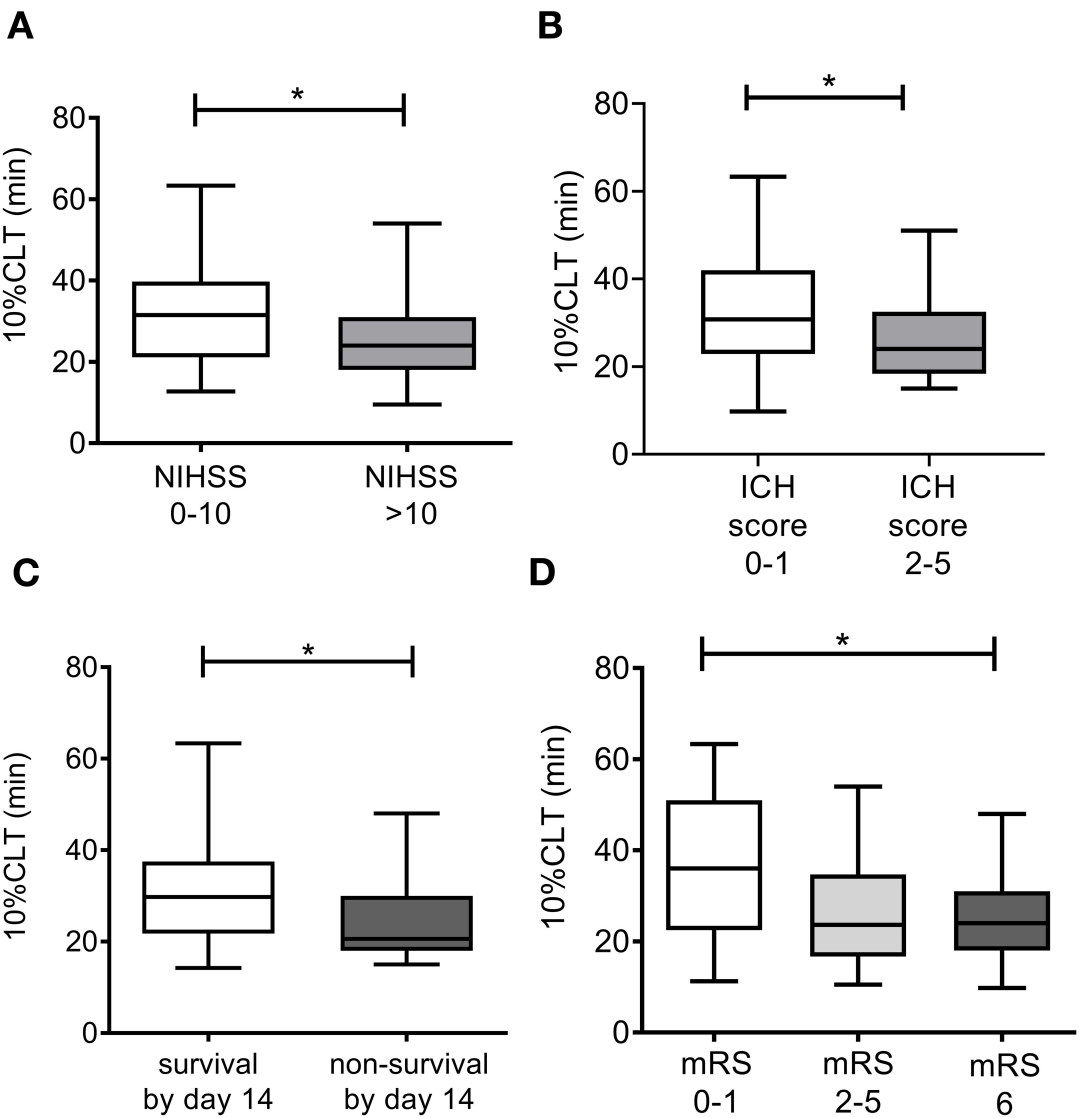
Associations of the 10%CLT parameter of the modified clot lysis assay (mCLA) with stroke severity on admission **(A)**, ICH score calculated on admission **(B)**, survival by day 14 **(C)**, and the modified Rankin Scale (mRS) on day 90 **(D)**. mCLA is performed in the presence of cell-free DNA and histones. Box and whisker plots indicate median, interquartile range, and total range. 10% CLT: 10% clot lysis time, ICH, intracerebral hemorrhage; NIHSS, National Institutes of Health Stroke Scale; mRS, modified Rankin Scale. *p < 0.05.

As expected, clot lysis parameters (max. absorbance, 10%CLT, 50% CLT, and AUC) became significantly prolonged in the total cohort when cfDNA and histones were added to the sample solutions (Table 2). Patients with ICH showed significantly shorter clot lysis times as compared to a healthy reference group (Supplementary Table 1), indicating faster fibrinolysis, that was independent of the addition of cfDNA and histones. However, stroke severity and outcomes showed no association with the conventional CLA in the absence of DNA and histones (Supplementary Tables 2–4). Stroke severity and outcomes showed no association with the difference obtained between mCLA and CLA parameters (data not shown). On the contrary, patients with more severe stroke (NIHSS > 10) showed significantly shorter clot lysis (10%CLT) in the modified test as compared to patients with milder stroke (NIHSS 0–10) (Figure 1A). Similarly, significantly shorter clot lysis was observed using the mCLA in patients with higher ICH score (2–5) as compared to those with ICH 0–1 (Figure 1B). Key proteins of the fibrinolytic system (plasminogen, α2-PI, and FXIII activity) showed significant correlation with the maximal absorbance parameter of the conventional and mCLA, moreover, plasminogen activity showed significant correlation with most parameters of both assays (Supplementary Table 5). However, stroke severity (data not shown) and outcomes showed no association with any of the tested coagulation or fibrinolysis protein activity levels (Tables 3, 4).

**Table 2:** Clot lysis assay (CLA) and modified CLA (mCLA)^*^ parameters in the total cohort.

	**CLA**	**mCLA**	***p***
Max. absorbance (OD)	1.41 (1.30–1.59)	1.42 (1.32–1.60)	0.001
Time to max. absorbance (min)	10.5 (9.0–14.0)	11.5 (8.0–15.0)	0.906
10%CLT (min)	23.5 (15.5–33.0)	25.5 (18.5–35.0)	0.023
50%CLT (min)	34.5 (24.5–44.0)	35.5 (28.0–49.5)	0.012
90%CLT (min)	76.0 (66.0–87.0)	75.0 (68.0–87.0)	0.254
CLA AUC (OD^*^min)	24.2 (18.4–28.3)	25.2 (19.6–30.0)	0.004

* *mCLA is performed in the presence of cell-free DNA and histones. Data are medians (interquartile ranges); cfDNA, cell-free DNA; CLA; clot lysis assay, 10%CLT, 10% clot lysis time; 50%CLT, 50% clot lysis time; 90%CLT, 90% clot lysis time; CLA AUC, clot lysis assay area under the curve*.

**Table 3:** Baseline clinical data and modified clot lysis assay (mCLA)^*^ parameters according to mortality by day 14.

	**Survival by day 14** ** (*n* = 63)**	**Non-survival by day 14 (*n* = 26)**	***p***
Age, y, mean ± SD	67 ± 12	71 ± 10	0.082
Male sex, *n* (%)	29 (46)	10 (39)	0.474
Stroke severity on admission, NIHSS, median (IQR)	11 (5–17)	19 (16–23)	<0.0001
**Cerebrovascular risk factors**, ***n*** **(%)**			
Arterial hypertension	59 (94)	26 (100)	0.552
Atrial fibrillation	7 (11)	3 (12)	0.999
Diabetes mellitus	24 (38)	14 (54)	0.191
Hyperlipidemia	34 (54)	14 (54)	0.932
Active smoker	14 (22)	2 (8)	0.032
BMI, kg/m^2^, median (IQR)	27.1 (23.3–32.4)	26.9 (25.4–30.6)	0.810
**Laboratory measurements on admission, median (IQR)**			
INR	0.96 (0.93–1.00)	1.02 (0.96–1.68)	0.009
APTT, s	27.7 (25.4–30.1)	28.3 (25.1–36.5)	0.430
WBC, G/L	8.6 (6.5-11.2)	9.3 (6.9-12.0)	0.538
Platelet count, G/L	238 (172–283)	203 (167–242)	0.049
Serum glucose, mmol/L	7.3 (5.8–9.7)	8.2 (6.5–11.4)	0.094
hsCRP, mg/L	3.2 (1.2–7.1)	2.4 (1.3–3.8)	0.373
Creatinine, μmol/L	69 (60-82)	76 (62-95)	0.326
Fibrinogen, mg/ml	4.0 (3.2–4.6)	3.4 (2.9–4.2)	0.086
Plasminogen activity (%)	112 (97-123)	107 (103-114)	0.212
α2- plasmin inhibitor activity (%)	107 (99–113)	105 (94–114)	0.422
Factor XIII activity (%)	167 (147–175)	161.5 (132–177)	0.414
**mCLA parameters**			
Maximal absorbance (OD)	1.48 ± 0.25	1.41 ± 0.21	0.374
Time to maximal absorbance (min)	11.5 (8.0–15.0)	10.0 (8.0–14.0)	0.724
10%CLT (min)	30.0 (22.0–37.5)	20.5 (18.0–30.0)	0.037
50%CLT (min)	39.0 (28.0–51.0)	34.0 (27.5–45.5)	0.491
90%CLT (min)	76.5 (69.0–87.0)	74.0 (61.0–82.5)	0.179
CLA AUC (OD^*^min)	25.6 (19.8–31.5)	24.1 (20.3–28.5)	0.459

* *mCLA is performed in the presence of cell-free DNA and histones. Data are means ± SD or medians (interquartile ranges). 10%CLT, 10% clot lysis time; 50%CLT, 50% clot lysis time; 90%CLT, 90% clot lysis time; CLA, clot lysis assay; CLA AUC, clot lysis assay area under the curve; APTT, activated partial thromboplastin time; BMI, body mass index; hsCRP, high sensitivity C-reactive protein measurement; INR, international normalized ratio*.

**Table 4:** Baseline clinical data and modified clot lysis assay (mCLA)^*^ parameters according to long-term functional outcomes at 90 days post-event.

	**mRS 0–1 (*n* = 15)**	**mRS 2–5 (*n* = 32)**	**mRS 6 (*n* = 39)**	***p***
Age, y, mean ± SD	64 ± 12	67 ± 13	71 ± 10	0.054
Male sex, *n* (%)	10 (67)	21 (66.0)	28 (72)	0.920
Stroke severity on admission, NIHSS, median (IQR)	5 (4–6)	13 (10–17)	19 (15–23)	<0.0001
				<0.0001^§^
				<0.001^#^
				0.015^†^
**Cerebrovascular risk factors**, ***n*** **(%)**				
Arterial hypertension	13 (87)	32 (100)	39 (100)	0.060
Atrial fibrillation	0 (0)	5 (16)	5 (13)	0.283
Diabetes mellitus	6 (40)	12 (38)	20 (51)	0.541
Hyperlipidemia	11 (73)	14 (44)	23 (59)	0.095
Active smoker	5 (33)	6 (19)	4 (10)	0.109
BMI, kg/m^2^, median (IQR)	23.6 (21.5–26.0)	30.2 (25.0–33.2)	27.1 (25.4–31.6)	0.012
				0.037^§^
				0.011^#^
**Laboratory measurements on admission, median (IQR)**				
INR	0.96 (0.94–0.99)	0.95 (0.91–0.99)	1.01 (0.94–1.21)	0.029
				0.033^†^
APTT, s	27.6 (25.7–29.6)	28.0 (24.8–30.4)	27.9 (25.4–33.3)	0.822
WBC, G/L	6.9 (6.5–9.4)	8.8 (7.6–12.3)	8.8 (6.7–11.4)	0.315
Platelet count, G/L	250 (158–273)	253 (209–288)	200 (168–243)	0.032
				0.027^#^
Serum glucose, mmol/L	7.4 (5.5–11.2)	7.3 (6.0–9.8)	7.7 (6.3–11.2)	0.563
hsCRP, mg/L	1.5 (0.7–4.1)	4.2 (1.3–8.1)	2.7 (1.2–7.0)	0.193
Creatinine, μmol/L	70 (54–88)	68 (55–83)	72 (64–88)	0.478
Fibrinogen, mg/ml	4.0 (3.2–4.3)	3.8 (3.3–4.6)	3.5 (3.0–4.5)	0.339
Plasminogen activity (%)	111 (93–123)	113 (100–132)	107 (103–117)	0.456
α2-plasmin inhibitor activity (%)	103 (99–109)	112 (103–115)	105 (96–114)	0.135
Factor XIII activity (%)	160 (138–175)	174 (160–180)	165 (141–178)	0.071
**mCLA parameters**				
Maximal absorbance (OD)	1.37 (1.3–1.6)	1.50 (1.4–1.6)	1.42 (1.3–1.6)	0.454
Time to maximal absorbance (min)	15.0 (12.0–16.5)	10.5 (8.0–14.0)	9.5 (7.5–14.5)	0.039
				0.047^§^
10%CLT (min)	36.0 (22.5–51.0)	23.5 (17.8–36.0)	22.5 (18.0–30.5)	0.027
				0.032^§^
50%CLT (min)	48.0 (42.0–63.0)	32.0 (28.0–49.5)	34.5 (27.0–45.0)	0.041
				0.043^§^
90%CLT (min)	81.0 (68.0–90.0)	75.0 (69.0–85.5)	75.0 (68.0–87.0)	0.490
CLA AUC (OD^*^min)	29.4 (23.7–34.5)	24.6 (18.8–29.7)	24.4 (19.5–29.3)	0.149

* *mCLA is performed in the presence of cell-free DNA and histones. Data are means ± SD or medians (interquartile ranges). 10%CLT, 10% clot lysis time; 50%CLT, 50% clot lysis time; 90%CLT, 90% clot lysis time; APTT, activated partial thromboplastin time; BMI, body mass index; hsCRP, high sensitivity C-reactive protein measurement; CLA, clot lysis time; CLA AUC, clot lysis assay area under the curve; INR, international normalized ratio; mRS, modified Rankin Scale*.

§ *mRS 0–1 vs. mRS 6 (ANOVA, Bonferroni post-hoc-test or Kruskal-Wallis, Dunn's post-hoc-test)*.

† *mRS 2–5 vs. mRS 6 (ANOVA, Bonferroni post-hoc-test)*.

# *mRS 0–1 vs. mRS 2-5 (ANOVA, Bonferroni post-hoc-test or Kruskal-Wallis, Dunn's post-hoc-test)*.

**Figure 2:**
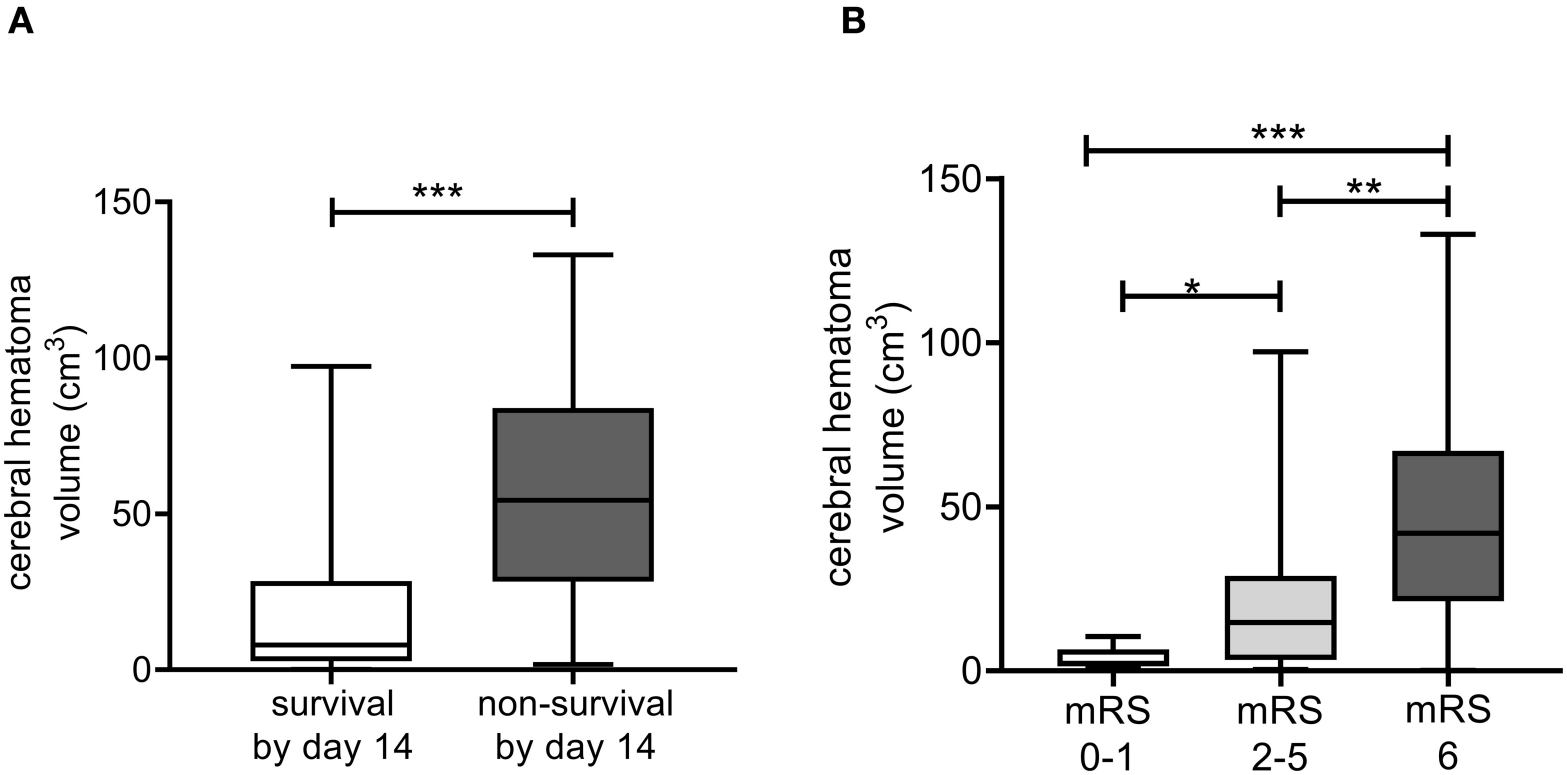
Associations of the estimated cerebral hematoma volume as calculated from on admission CT scans with patient survival by day 14 **(A)**, and the modified Rankin Scale (mRS) on day 90 **(B)**. Box and whisker plots indicate median, interquartile range, and total range. mRS, modified Rankin Scale. *p < 0.05, **p < 0.01, ***p < 0.001.

**Figure 3:**
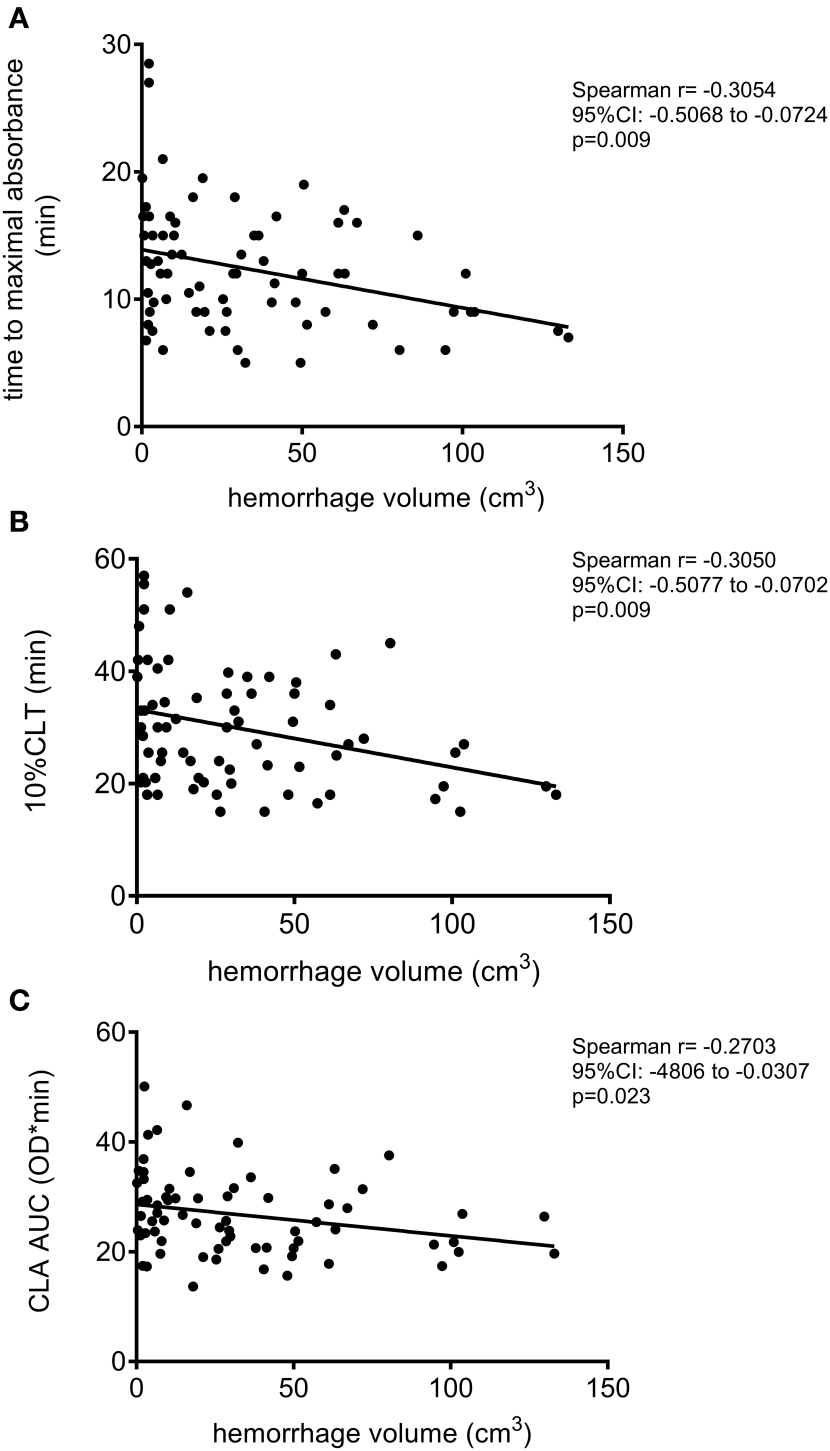
Correlation between the modified clot lysis assay (mCLA) parameters and estimated intracerebral hematoma volume of the ICH patients. mCLA is performed in the presence of cell-free DNA and histones. Correlation between estimated intracerebral hemorrhage volume and time to maximal absorbance parameter **(A)**, 10% clot lysis time (10%CLT) **(B)**, and clot lysis assay area under the curve (CLA AUC) **(C)** are depicted.

Mortality by day 14 was associated with significantly shorter 10%CLT of the mCLA (Table 3 and Figure 1C). The median 10%CLT was 9.5 min shorter in those patients who died by day 14 as compared to those who survived (*p* = 0.037). Besides CLA parameters, admission NIHSS, INR, smoking and platelet count showed association with mortality by day 14. Similarly to short-term outcomes by day 14, results of long-term functional outcomes showed significant association with parameters of the modified assay (Table 4 and Figure 1D). Those patients, who died or had unfavorable outcomes (mRS ≥ 2) by the end of the 3rd month, demonstrated significantly shorter mCLA parameters on admission as compared to those with good functional outcomes. Besides mCLA parameters, admission NIHSS, BMI, INR and platelet count were associated with outcomes by day 90. In addition, estimated hemorrhage volume on admission showed strong association with day 14 and day 90 mortality (Figure 2). Notably, mCLA parameters correlated significantly with estimated intracerebral hemorrhage volume (Figure 3). mCLA parameters indicating faster clot formation and lysis (shorter 10%CLT and time to maximal absorbance parameters, lower CLA AUC) showed significant association with larger hemorrhage volumes.

**Figure 4:**
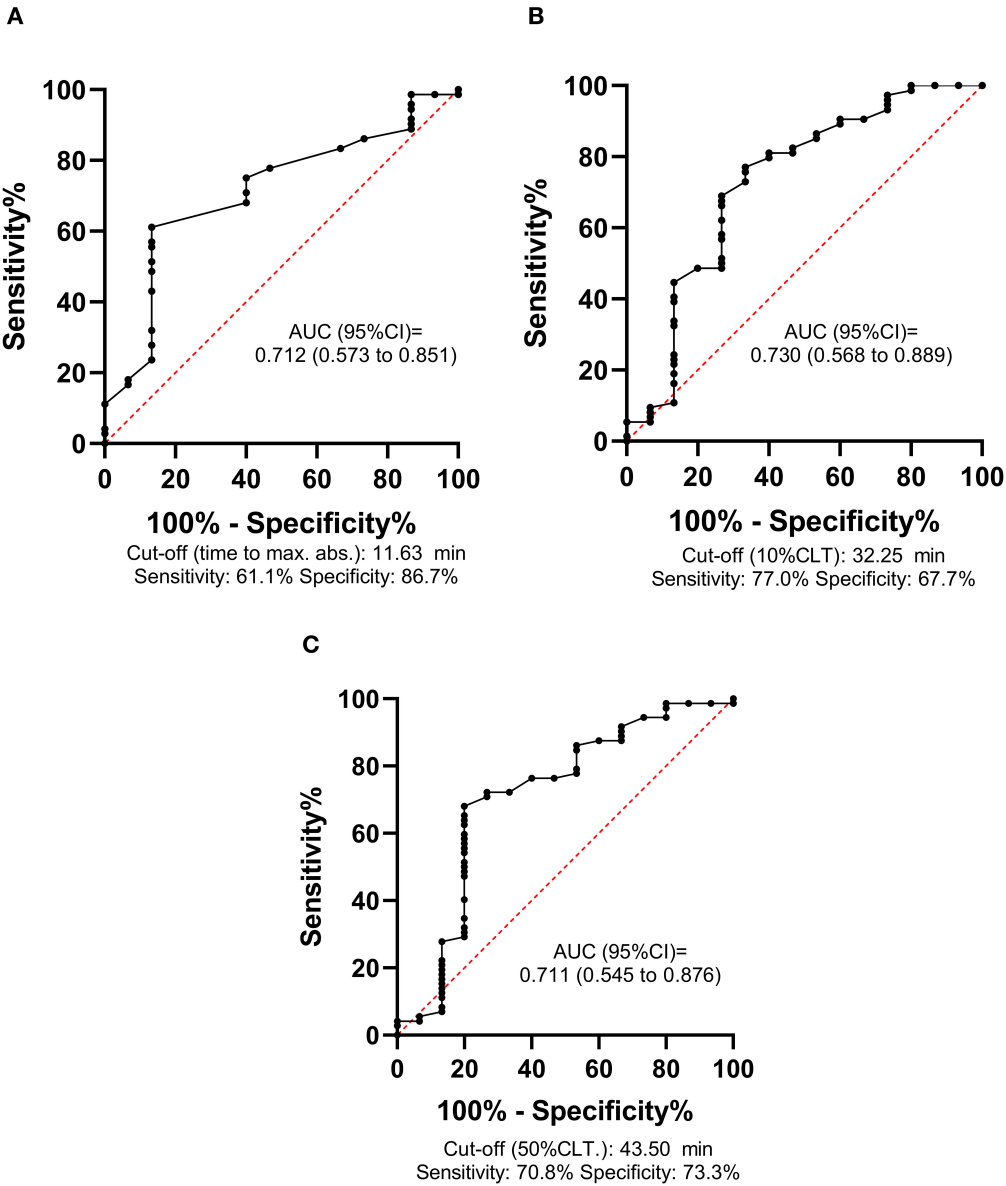
Receiver operator characteristic (ROC) curves of the modified clot lysis assay (mCLA) parameters for predicting long-term functional outcomes (mRS 0–1 vs. 2–6) of intracerebral hemorrhage stroke patients. mCLA is performed in the presence of cell-free DNA and histones. ROC curve and descriptive statistics including best cut-off value as determined by the Youden index are depicted for time to maximal absorbance parameter **(A)**, 10% clot lysis time (10%CLT) **(B)**, 50% clot lysis time (50%CLT) **(C)**.

**Figure 5:**
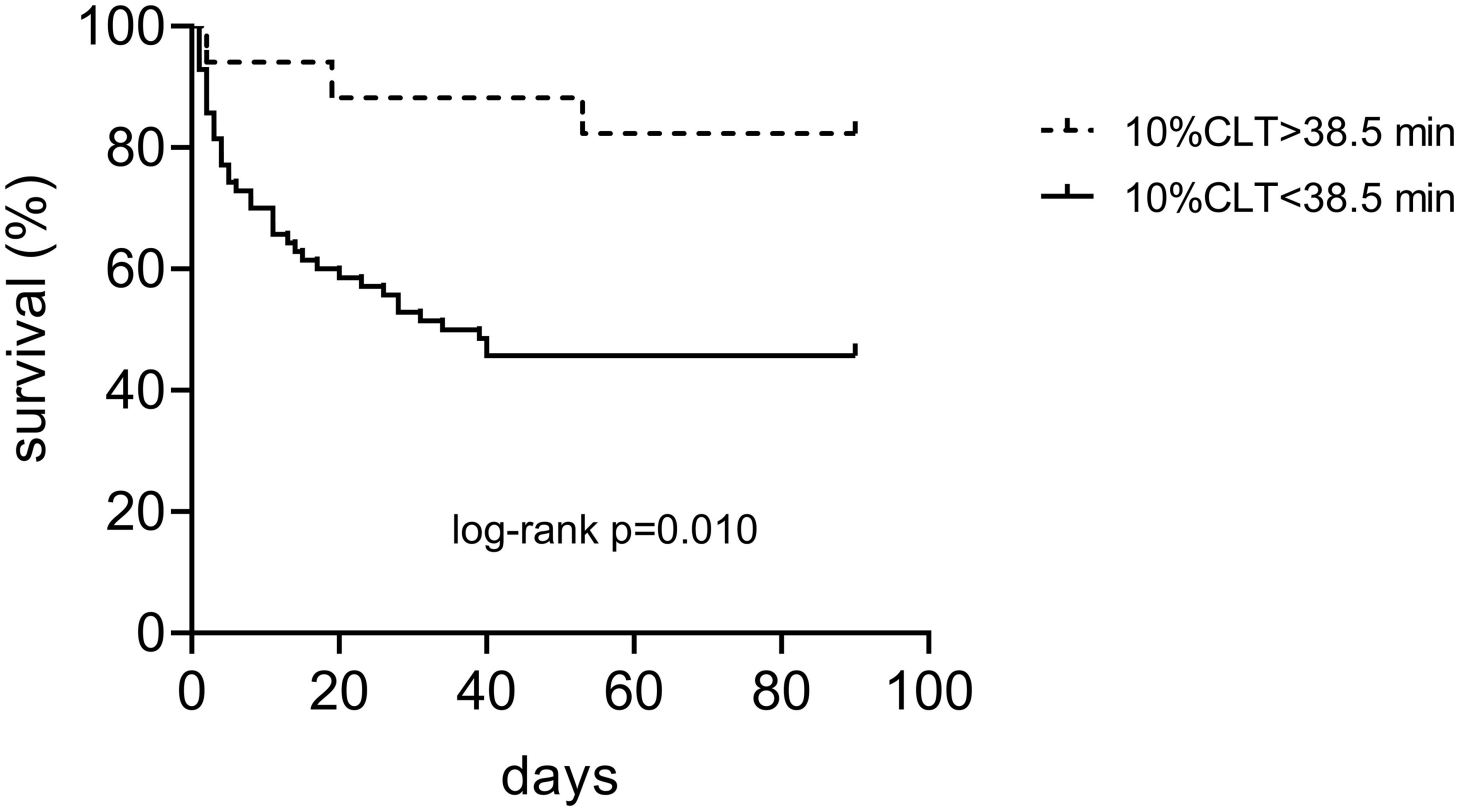
Kaplan-Meier survival curves of patients with spontaneous intracerebral hemorrhage according to the result of the modified clot lysis assay on admission (10%CLT below and above the limit of 38.5 min).

ROC analysis was performed for all outcomes to investigate the diagnostic performance of mCLA parameters (Figure 4). The best AUC of ROC was 0.73 (95%CI: 0.57–0.89) for the parameter 10%CLT for predicting mRS 0–1 as outcome (Figure 4B). Based on the optimal threshold value as defined by the Youden index (32.25 min), best sensitivity and specificity was provided by the 10%CLT parameter (77.0 and 67.7%, respectively, Figure 4B). When performing ROC analysis for mortality by day 14 and day 90, similar optimal threshold values were defined (10%CLT cut-off: >38.5 min for 90 day survival, curves not shown). In a Kaplan-Meier survival analysis, those patients who presented with a 10%CLT result of >38.5 min on admission showed significantly better survival as compared to those with shorter clot lysis results (*p* = 0.010; Figure 5).

Using binary backward logistic regression models (including age, sex, NIHSS on admission, hypertension, INR, platelet count, smoking status, cerebral hemorrhage volume, 10%CLT and BMI), 10%CLT of the mCLA did not prove to be an independent predictor of mortality by 14 days and 90 days post-event (Table 5). On the other hand, a binary backward logistic regression model (including age, sex, NIHSS on admission, INR, BMI, platelet count, 10%CLT, hemorrhage volume, hypertension) revealed that a shorter 10%CLT of the mCLA (<32.25 min) is a significant, independent predictor of unfavorable long-term functional outcome (mRS ≥ 2) (OR: 6.14, 95%CI: 1.11–34.02, *p* = 0.038) (Table 5).

**Table 5:** Independent predictors of outcomes in the studied cohort.

	**OR**	**95%CI**	***p***
**Mortality by day 14^§^**			
NIHSS on admission	1.20	1.09–1.32	0.0001
INR	3.54	1.06–11.85	0.041
**Mortality by day 90 (mRS 0–5 vs. mRS 6)^#^**			
NIHSS on admission	1.17	1.07–1.28	0.001
INR	2.02	0.71–5.75	0.190
mCLA 10%CLT <38.5 min	3.69	0.84–16.15	0.083
**Unfavorable long-term outcome (mRS 0–1 vs. mRS 2–6)^†^**			
NIHSS on admission	1.57	1.21–2.05	0.001
mCLA 10%CLT <32.25 min	6.14	1.11–34.02	0.038

*Last step of backward multiple regression analysis is provided*.

§ *Backward multiple regression model included age, sex, NIHSS on admission, hypertension, INR, platelet count, smoking status, hemorrhage volume, mCLA 10%CLT (threshold: <30.25 min)*.

# *Backward multiple regression model included age, sex, NIHSS on admission, hypertension, BMI, INR, platelet count, hemorrhage volume, mCLA 10%CLT (threshold: <38.5 min)*.

† *Backward multiple regression model included age, sex, NIHSS on admission, hypertension, BMI, INR, platelet count, hemorrhage volume, mCLA 10%CLT (threshold: <32.25 min)*.

*95%CI, 95% confidence interval; 10%CLT, 10% clot lysis time; INR, international normalized ratio; mCLA, modified clot lysis assay performed in the presence of cell-free DNA and histones; NIHSS, National Institutes of Health Stroke Scale; OR, odds ratio*.

## Discussion

To our knowledge, the current study shows for the first time that a CLA might be a promising tool to predict the outcome of intracerebral hemorrhagic stroke. Despite the clear benefit of diagnostic tests with acceptable predictive value regarding outcomes in acute ICH stroke patients, surprisingly few studies are available on this topic. Here we show that mCLA parameters of patients on admission correlate with the estimated size of hematoma on admission, which is an important predictor of outcomes. Shorter clot formation and lysis times, indicating faster break-down of the newly formed clot were associated with larger hematoma volume, more severe stroke, and worse outcomes in this cohort. These results are in line with previous studies revealing that an elevated D-dimer, indicating a more extensive break-down of clots, is associated with adverse outcomes in patients with ICH (5–7).

Our results clearly show a potential effect of the distortion of fibrinolytic balance on the evolution of the intracerebral hematoma. Fibrinogen levels were within the normal range in this cohort, suggesting that fibrinolytic factors rather than fibrinogen itself might be involved in this process. On the other hand, FXIII activity, plasminogen and α2-PI activity did not show an association with outcomes, suggesting the presence of other fibrinolytic alterations. Gaining knowledge on the factors that drive the enlargement or the dissolution of the bleeding are potentially important when designing future pharmacological therapies. As a first step, it is crucial to understand the underlying pathomechanism leading to poor outcomes in patients, and adequate diagnostic tools are a pre-requisite of such approaches.

The CLA is a method that has been shown to be potentially useful to predict outcomes in a wide range of pathologies where the fibrinolytic balance has been tilted (14, 18–25). The assay has a list of benefits and limitations, and most importantly, optimal conditions of the test have not been clearly defined, as yet. An undisputable advantage of the assay is being a global test of clot formation and lysis, thus saving efforts to determine the levels of individual factors of fibrinolysis using laborous and time-consuming methods. On the other hand, despite attempts to standardize the test, analytical challenges remain to be elaborated. In our study, we aimed to improve the diagnostic performance of the assay by incorporating the effect of cellular components that are known to influence fibrinolysis. In our assay conditions of mCLA, optimal concentrations of cfDNA and histones were adapted from previous *in vitro* studies using purified fibrinogen and various concentrations of cfDNA and histones, testing their combined effect on fibrinolysis (16). It must be emphasized, that our primary goal was to find assay conditions where fibrinolysis kinetics are optimally influenced by the addition of cfDNA and histones. The mechanisms behind the observed effect of cfDNA and histones resulting in prolongation of clot lysis are complex (14, 37). Among others, the addition of cfDNA promotes the formation of densely packed networks of thick fibers less susceptible to plasmin digestion, while the addition of histones competitively inhibits plasmin and delays fibrinolysis. Assay conditions of the mCLA represent an increased pool of DNA and histones, likely to be present during *in vivo* clot formation, as published previously (16, 24). The source of cfDNA and histones in the intracerebral compartment could also originate from tissue death (38) and might have an important modulatory effect on coagulation and fibrinolysis. Using such assay conditions in this study, the diagnostic performance of the assay was found to be particularly good to predict unfavorable long-term outcomes. In a binary logistic regression model, a shorter 10%CLT of the mCLA (<32.25 min) proved to be an independent predictor of unfavorable long-term outcomes (mRS ≥ 2).

The CLA is a test with the potential to represent *in vivo* global hemostasis and fibrinolysis upon a few modifications. In future studies, assay conditions might be further improved and standardized, allowing direct comparison between laboratories. The ultimate goal in the development and standardization of the CLA and related global fibrinolytic assays will be to generate standardized assay conditions that lead to highly sensitive and specific tests that aid clinical decision making, while allowing interlaboratory comparison of larger datasets. Clinical studies testing the utility of various fibrinolysis tests in predicting the outcome of thrombotic events are scarcely found in the literature as yet. Our study, similarly to few previous studies testing fibrinolysis in patient cohorts, is a hypothesis-generating study to obtain insights whether fibrinolytic abnormalities are associated with poor outcomes in ICH patients (39–41). Further long-term follow up studies are warranted to verify our results and optimize assay conditions.

## Conclusions

Parameters of the mCLA correlate well with ICH bleeding volume and could suggest unfavorable outcomes in spontaneous, non-traumatic ICH. Future studies including large cohorts of patients with ICH are warranted to further study the relevance of fibrinolysis alterations in the evolution of intracerebral hematoma and patient outcomes. Further modifications of the test might allow better diagnostic performance and easier implementation, which might be necessary for its potential clinical utilization in the future.

## Limitations

Results of the present study should be interpreted in the context of its limitations and strengths. The sample size is limited, however, as compared to other published prospective studies including consecutive patients with non-traumatic, spontaneous ICH, involving the measurement of hemostasis biomarkers from admission samples, it is among the largest studies as yet. Nevertheless, results presented here must be confirmed and validated by larger studies. The study was single-centered, which contributed to the limited sample size, but it had the advantages of uniform sample handling and patient care, and the major benefit that few patients were lost to follow-up. During follow-up only one patient acquired SARS-CoV-2 infection post-event, and in this case long-term follow-up results were excluded.

## Data Availability Statement

The raw data supporting the conclusions of this article will be made available by the authors, without undue reservation.

## Ethics Statement

The studies involving human participants were reviewed and approved by Institutional Ethics Committee of the University of Debrecen and the Ethics Committee of the National Medical Research Council. The patients/participants provided their written informed consent to participate in this study.

## Author Contributions

RO-K collected clinical samples, performed experiments, and analyzed and interpreted data. TÁ, IF, KF, MH, and JT collected clinical data and analyzed and interpreted data. FS collected clinical samples and performed experiments. LC designed the research and analyzed and interpreted the data. ZB analyzed and interpreted the data and wrote the manuscript. All authors have read and approved the final manuscript.

## Conflict of Interest

The authors declare that the research was conducted in the absence of any commercial or financial relationships that could be construed as a potential conflict of interest.

## Supplementary Material

Representative CLA curves and reference parameters of healthy controls as compared to patients are provided as Supplementary Figure 1 and Supplementary Table 1. Associations between parameters of the conventional CLA and stroke severity or outcomes are shown in Supplementary Tables 2–4. Correlation between fibrinolysis parameters and clot lysis assay parameters in the absence (CLA) or presence of cfDNA and histones (mCLA) are provided in Supplementary Table 5.

## References

[1] Pinho J Costa AS Araujo JM Amorim JM Ferreira C. Intracerebral hemorrhage outcome: a comprehensive update. J Neurol Sci. (2019) 398:54–66. 10.1016/j.jns.2019.01.013 30682522

[2] Qureshi AI Tuhrim S Broderick JP Batjer HH Hondo H Hanley DF. Spontaneous intracerebral hemorrhage. N Engl J Med. (2001) 344:1450–60. 10.1056/NEJM200105103441907 11346811

[3] Zakai NA Olson NC Judd SE Kleindorfer DO Kissela BM Howard G. Haemostasis biomarkers and risk of intracerebral haemorrhage in the reasons for geographic and racial differences in stroke study. Thromb Haemost. (2017) 117:1808–15. 10.1160/TH17-03-0189 28692106 PMC6309529

[4] Quinones-Hinojosa A Gulati M Singh V Lawton MT. Spontaneous intracerebral hemorrhage due to coagulation disorders. Neurosurg Focus. (2003) 15:E3. 10.3171/foc.2003.15.4.3 15344896

[5] Chiu CC Li YN Lin LJ Hsiao CT Hsiao KY Chen IC. Serum D-dimer as a predictor of mortality in patients with acute spontaneous intracerebral hemorrhage. J Clin Neurosci. (2012) 19:810–3. 10.1016/j.jocn.2011.08.032 22377638

[6] Hu X Fang Y Ye F Lin S Li H You C. Effects of plasma D-dimer levels on early mortality and long-term functional outcome after spontaneous intracerebral hemorrhage. J Clin Neurosci. (2014) 21:1364–7. 10.1016/j.jocn.2013.11.030 24631325

[7] Delgado P Alvarez-Sabin J Abilleira S Santamarina E Purroy F Arenillas JF. Plasma d-dimer predicts poor outcome after acute intracerebral hemorrhage. Neurology. (2006) 67:94–8. 10.1212/01.wnl.0000223349.97278.e0 16832084

[8] Lisman T de Groot PG Meijers JC Rosendaal FR. Reduced plasma fibrinolytic potential is a risk factor for venous thrombosis. Blood. (2005) 105:1102–5. 10.1182/blood-2004-08-3253 15466929

[9] Siudut J Natorska J Wypasek E Wiewiorka L Ostrowska-Kaim E Wisniowska-Smialek S. Impaired fibrinolysis in patients with isolated aortic stenosis is associated with enhanced oxidative stress. J Clin Med. (2020) 9:2002. 10.3390/jcm9062002 32630544 PMC7355626

[10] Gitto S Romanelli RG Cellai AP Lami D Vizzutti F Abbate R. Altered clot formation and lysis are associated with increased fibrinolytic activity in ascites in patients with advanced cirrhosis. Intern Emerg Med. (2020) 16:339–47. 10.1007/s11739-020-02375-3 32445164

[11] Pitkanen HH Karki M Niinikoski H Tanner L Nanto-Salonen K Pikta M. Abnormal coagulation and enhanced fibrinolysis due to lysinuric protein intolerance associates with bleeds and renal impairment. Haemophilia. (2018) 24:e312–21. 10.1111/hae.13543 30070418

[12] Bembenek JP Niewada M Siudut J Plens K Czlonkowska A Undas A. Fibrin clot characteristics in acute ischaemic stroke patients treated with thrombolysis: the impact on clinical outcome. Thromb Haemost. (2017) 117:1440–7. 10.1160/TH16-12-0954 28382369

[13] Cieslik J Mrozinska S Broniatowska E Undas A. Altered plasma clot properties increase the risk of recurrent deep vein thrombosis: a cohort study. Blood. (2018) 131:797–807. 10.1182/blood-2017-07-798306 29242187

[14] Varju I Kolev K. Networks that stop the flow: a fresh look at fibrin and neutrophil extracellular traps. Thromb Res. (2019) 182:1–11. 10.1016/j.thromres.2019.08.003 31415922

[15] Varju I Longstaff C Szabo L Farkas AZ Varga-Szabo VJ Tanka-Salamon A. DNA, histones and neutrophil extracellular traps exert anti-fibrinolytic effects in a plasma environment. Thromb Haemost. (2015) 113:1289–98. 10.1160/TH14-08-0669 25789443

[16] Longstaff C Varju I Sotonyi P Szabo L Krumrey M Hoell A. Mechanical stability and fibrinolytic resistance of clots containing fibrin, DNA, and histones. J Biol Chem. (2013) 288:6946–56. 10.1074/jbc.M112.404301 23293023 PMC3591605

[17] Brinkmann V Reichard U Goosmann C Fauler B Uhlemann Y Weiss DS. Neutrophil extracellular traps kill bacteria. Science. (2004) 303:1532–5. 10.1126/science.1092385 15001782

[18] Laridan E Denorme F Desender L Francois O Andersson T Deckmyn H. Neutrophil extracellular traps in ischemic stroke thrombi. Ann Neurol. (2017) 82:223–32. 10.1002/ana.24993 28696508

[19] Ducroux C Di Meglio L Loyau S Delbosc S Boisseau W Deschildre C. Thrombus neutrophil extracellular traps content impair tPA-induced thrombolysis in acute ischemic stroke. Stroke. (2018) 49:754–7. 10.1161/STROKEAHA.117.019896 29438080

[20] Hisada Y Grover SP Maqsood A Houston R Ay C Noubouossie DF. Neutrophils and neutrophil extracellular traps enhance venous thrombosis in mice bearing human pancreatic tumors. Haematologica. (2020) 105:218–25. 10.3324/haematol.2019.217083 31048354 PMC6939515

[21] Fuchs TA Brill A Wagner DD. Neutrophil extracellular trap (NET) impact on deep vein thrombosis. Arterioscler Thromb Vasc Biol. (2012) 32:1777–83. 10.1161/ATVBAHA.111.242859 22652600 PMC3495595

[22] Thalin C Hisada Y Lundstrom S Mackman N Wallen H. Neutrophil extracellular traps: villains and targets in arterial, venous, and cancer-associated thrombosis. Arterioscler Thromb Vasc Biol. (2019) 39:1724–38. 10.1161/ATVBAHA.119.312463 31315434 PMC6703916

[23] Valles J Lago A Santos MT Latorre AM Tembl JI Salom JB. Neutrophil extracellular traps are increased in patients with acute ischemic stroke: prognostic significance. Thromb Haemost. (2017) 117:1919–29. 10.1160/TH17-02-0130 28837206

[24] Fuchs TA Brill A Duerschmied D Schatzberg D Monestier M Myers DD Jr.. Extracellular DNA traps promote thrombosis. Proc Natl Acad Sci USA. (2010) 107:15880–5. 10.1073/pnas.1005743107 20798043 PMC2936604

[25] Geddings JE Mackman N. New players in haemostasis and thrombosis. Thromb Haemost. (2014) 111:570–4. 10.1160/TH13-10-0812 24573314 PMC4080798

[26] Kothari RU Brott T Broderick JP Barsan WG Sauerbeck LR Zuccarello M. The ABCs of measuring intracerebral hemorrhage volumes. Stroke. (1996) 27:1304–5. 10.1161/01.STR.27.8.1304 8711791

[27] Brott T Adams HP Jr. Olinger CP Marler JR Barsan WG Biller J. Measurements of acute cerebral infarction: a clinical examination scale. Stroke. (1989) 20:864–70. 10.1161/01.STR.20.7.864 2749846

[28] Hemphill JC III Bonovich DC Besmertis L Manley GT Johnston SC. The ICH score: a simple, reliable grading scale for intracerebral hemorrhage. Stroke. (2001) 32:891–7. 10.1161/01.STR.32.4.891 11283388

[29] van Swieten JC Koudstaal PJ Visser MC Schouten HJ van Gijn J. Interobserver agreement for the assessment of handicap in stroke patients. Stroke. (1988) 19:604–7. 10.1161/01.STR.19.5.604 3363593

[30] Banks JL Marotta CA. Outcomes validity and reliability of the modified Rankin scale: implications for stroke clinical trials: a literature review and synthesis. Stroke. (2007) 38:1091–6. 10.1161/01.STR.0000258355.23810.c6 17272767

[31] Talens S Malfliet JJ Rudez G Spronk HM Janssen NA Meijer P. Biological variation in tPA-induced plasma clot lysis time. Thromb Haemost. (2012) 108:640–6. 10.1160/TH12-02-0124 22836204

[32] Pieters M Philippou H Undas A de Lange Z Rijken DC Mutch NJ Subcommittee on factor X fibrinogen the subcommittee on F. An international study on the feasibility of a standardized combined plasma clot turbidity and lysis assay: communication from the SSC of the ISTH. J Thromb Haemost. (2018) 16:1007–12. 10.1111/jth.14002 29658191

[33] Posch F Hofer S Thaler J Hell L Konigsbrugge O Grilz E. *Ex vivo* properties of plasma clot formation and lysis in patients with cancer at risk for venous thromboembolism, arterial thrombosis, and death. Transl Res. (2020) 215:41–56. 10.1016/j.trsl.2019.08.009 31525325 PMC7332340

[34] Longstaff C Subcommittee on Fibrinolysis. Development of Shiny app tools to simplify and standardize the analysis of hemostasis assay data: communication from the SSC of the ISTH. J Thromb Haemost. (2017) 15:1044–6. 10.1111/jth.13656 28304129

[35] Chesher D. Evaluating assay precision. Clin Biochem Rev. (2008) 29(Suppl. 1):S23–6. PMC2556577 18852851

[36] Clinical and Laboratory Standards Institute. Evaluation of Precision of Quantitative Measurement Procedures; Approved Guideline-Third Edition. CLSI Document EP05-A3. Wayne, PA: Clinical and Laboratory Standards Institute (2014).

[37] Locke M Longstaff C. Extracellular histones inhibit fibrinolysis through noncovalent and covalent interactions with fibrin. Thromb Haemost. (2020) 121:464–76. 10.1055/s-0040-1718760 33131044 PMC7982298

[38] Rodrigues Filho EM Simon D Ikuta N Klovan C Dannebrock FA Oliveira de Oliveira C. Elevated cell-free plasma DNA level as an independent predictor of mortality in patients with severe traumatic brain injury. J Neurotrauma. (2014) 31:1639–46. 10.1089/neu.2013.3178 24827371 PMC4171115

[39] Fraczek P Krzysztofik M Stanisz A Undas A. Clinical outcomes and plasma clot permeability and lysability in patients with venous thromboembolism on rivaroxaban: a cohort study. Pol Arch Intern Med. (2019) 129:377–85. 10.20452/pamw.14824 31063157

[40] Undas A. Altered fibrin clot properties and fibrinolysis in patients with atrial fibrillation: practical implications. Europace. (2020) 22:185–94. 10.1093/europace/euz271 31625555

[41] Kleinegris MF Konings J Daemen JW Henskens Y de Laat B Spronk HMH. Increased clot formation in the absence of increased thrombin generation in patients with peripheral arterial disease: a case-control study. Front Cardiovasc Med. (2017) 4:23. 10.3389/fcvm.2017.00023 28473975 PMC5397513

