# Supplementary material for: A Modified in vitro Clot Lysis Assay Predicts Outcomes in Non-traumatic Intracerebral Hemorrhage Stroke Patients—The IRONHEART Study
Source: Front Neurol. 2021 Apr 20;12:613441. doi: 10.3389/fneur.2021.613441 (PMC8093390; doi:10.3389/fneur.2021.613441)
Supplement: Supplementary file 1 [file Data_Sheet_1.docx]

Supplementary Material

**Supplementary Figure 1. Clot lysis assay performed in the absence (CLA) or presence of cell free DNA and histones (modified CLA).** Clot lysis curves of CLA (Figure 1A) and modified CLA (Figure 1B) together with the main parameters of the curves are shown. Various colors represent parallel measurements of the same representative sample. All samples were run in quadruplicates. AUC; area under the curve, 10%CLT; 10% clot lysis time, 50%CLT; 50% clot lysis time, 90%CLT; 90% clot lysis time.

**
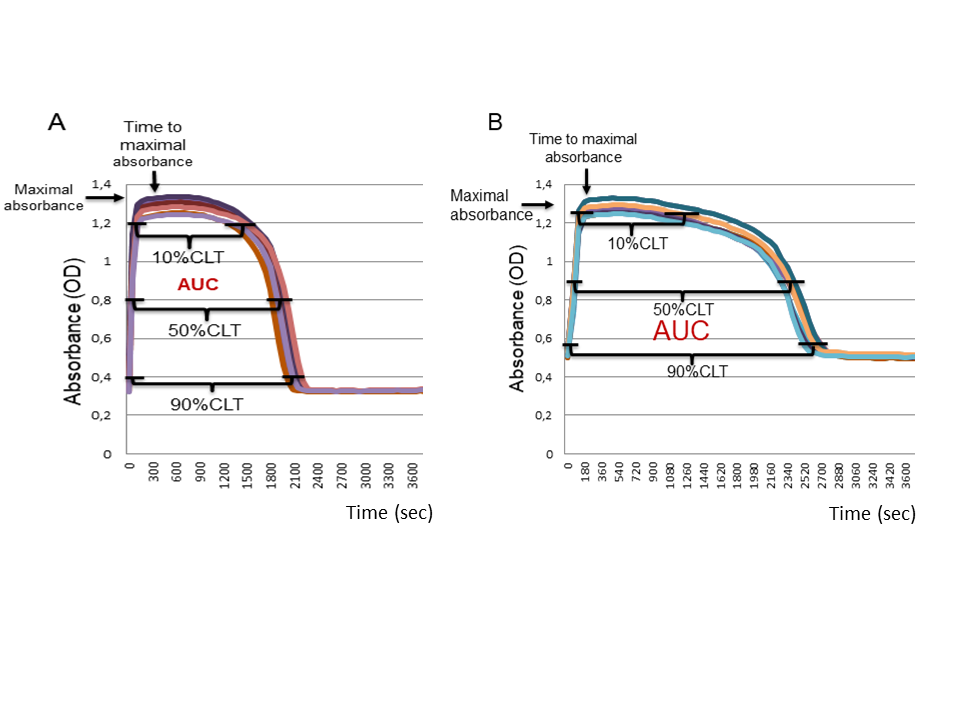
**

**Supplementary Table 1.** Clot lysis assay (CLA) and modified clot lysis assay (mCLA)* parameters in healthy reference individuals vs. patients with intracranial hemorrhage (ICH)

|  | **Healthy reference individuals** | **ICH patients** | **p** |
| --- | --- | --- | --- |
| Number of patients, n | 29 | 89 |  |
| Age, y, mean±SD | 44 (±11.7) | 68 (±11.6) | <0.0001 |
| Male sex, n (%) | 8 (27.6) | 57 (64.0) | 0.017 |
| Clot lysis assay (CLA) parameters | | | |
| maximal absorbance (OD) | 1.3 (1.2-1.4) | 1.4 (1.3-1.6) | 0.006 |
| time to maximal absorbance (min) | 18.5 (13.0-29.0) | 10.5 (9.0-14.0) | <0.0001 |
| 10%CLT (min) | 49.0 (14.5-60.0) | 23.5 (15.5-33.0) | 0.006 |
| 50%CLT (min) | 73.55 (59.5-102.0) | 34.5 (24.5-44.0) | <0.0001 |
| 90%CLT (min) | 106.0 (85.0.0-136.5) | 76.0 (66.0-87.0) | <0.0001 |
| CLA AUC (OD*min) | 41.0 (34.5-47.0) | 24.2 (18.4-28.3) | <0.0001 |
| Modified clot lysis assay (mCLA) parameters | | | |
| maximal absorbance (OD) | 1.32±0.15 | 1.4 (1.3-1.6) | 0.002 |
| time to maximal absorbance (min) | 24.0 (16.0-34.5) | 11.5 (8.0-15.0) | <0.0001 |
| 10%CLT (min) | 57.0 (21.5-71.5) | 25.5 (18.5-35.0) | 0.0001 |
| 50%CLT (min) | 89.0 (70.0-111.5) | 35.5 (28.0-49.5) | <0.0001 |
| 90%CLT (min) | 119.0 (100.0-145.5) | 75.0 (68.0-87.0) | <0.0001 |
| CLA AUC (OD*min) | 43.0 (37.9-51.4) | 25.2 (19.6-30.0) | <0.0001 |

*mCLA is performed in the presence of cell-free DNA and histones. Data are means±SD or medians (interquartile ranges). CLA, clot lysis assay; CLA AUC, clot lysis assay area under the curve; mCLA, modified clot lysis assay; 10%CLT, 10% clot lysis time; 50%CLT, 50% clot lysis time; 90%CLT, 90% clot lysis time; ICH, intracerebral hemorrhage

**Supplementary Table 2.** Clot lysis assay (CLA) parameters according to stroke severity (NIHSS) on admission.

|  | **NIHSS**  **0-10** | **NIHSS**  **>10** | **p** |
| --- | --- | --- | --- |
| maximal absorbance | 1.51±0.28 | 1.42 ±0.21 | 0.081 |
| time to maximal absorbance (min) | 12.0 (9.0-16.5) | 10.0 (8.0-13.5) | 0.101 |
| 10%CLT (min) | 27.0 (18.0-35.5) | 22.0 (15.5-33.0) | 0.169 |
| 50%CLT (min) | 39.0 (26.0-45.5) | 31.5 (24.5-45.0) | 0.430 |
| 90%CLT (min) | 77.0 (57.5-87.0) | 76.0 (69.0-87.0) | 0.759 |
| CLA AUC (OD*min) | 24.4 (20.5-31.7) | 24.0 (18.2-28.1) | 0.284 |

Data are means ±SD or medians (interquartile ranges). 10%CLT, 10% clot-lysis time; 50%CLT, 50% clot-lysis time; 90%CLT, 90% clot-lysis time; CLA, clot lysis assay; CLA AUC, clot lysis assay area under the curve, NIHSS, National Institutes of Health Stroke Scale

**Supplementary Table 3.** Clot lysis assay (CLA) parameters according to mortality by day 14

|  | **Survival by**  **day 14**  **(n=61)** | | **Non-survival by day 14**  **(n=26)** | **p** |
| --- | --- | --- | --- | --- |
| maximal absorbance | | 1.40±0.25 | 1.41±0.21 | 0.318 |
| time to maximal absorbance (min) | | 10.5 (9.0-13.0) | 11.0 (9.0-15.0) | 0.592 |
| 10%CLT (min) | | 24.0 (15.5-33.0) | 22.0 (16.5-35.0) | 0.874 |
| 50%CLT (min) | | 35.0 (26.5-43.0) | 38.0 (24.0-49.0) | 0.442 |
| 90%CLT (min) | | 75.0 (69.0-87.0) | 78 (62.0-84.0) | 0.773 |
| CLA AUC (OD*min) | | 24.1 (18.5-29.1) | 24.5 (18.3-28.1) | 0.987 |

Data are means ±SD or medians (interquartile ranges). 10%CLT, 10% clot-lysis time; 50%CLT, 50% clot-lysis time; 90%CLT, 90% clot-lysis time; CLA, clot lysis assay; CLA AUC, clot lysis assay area under the curve

**Supplementary Table 4.** Clot lysis assay (CLA) parameters according to long-term functional outcomes at 90 days post-event

|  | **mRS 0-1**  **(n=15)** | **mRS 2-5**  **(n=32)** | **mRS 6**  **(n=39)** | **p** |
| --- | --- | --- | --- | --- |
| maximal absorbance | 1.35  (1.3-1.7) | 1.40  (1.3-1.6) | 1.43  (1.3-1.6) | 0.904 |
| time to maximal absorbance (min) | 12.0  (8.5-16.5) | 9.5  (9.0-12.0) | 10.5  (9.0-15.0) | 0.589 |
| 10%CLT (min) | 28.5  (17.5-36.5) | 21.0  (15.0-27.0) | 22.0  (16.5-35.0) | 0.231 |
| 50%CLT (min) | 39.0  (27.5-48.5) | 29.0  (21.0-43.5) | 31.5  (25.0-48.0) | 0.176 |
| 90%CLT (min) | 81.0  (57.5-88.5) | 75.0  (69.0-87.0) | 77.0  (63.0-87.0) | 0.971 |
| CLA AUC (OD*min) | 24.5  (22.0-38.1) | 21.9  (17.8-29.3) | 24.1  (19.3-28.3) | 0.340 |

Data are medians (interquartile ranges). 10%CLT, 10% clot-lysis time; 50%CLT, 50% clot-lysis time; 90%CLT, 90% clot-lysis time; CLA, clot lysis assay; CLA AUC, clot lysis assay area under the curve, mRS; modified Rankin Scale

**Supplementary Table 5.**  Correlation between fibrinolysis parameters and clot lysis assay parameters in the absence (CLA) or presence of cell free DNA and histones (modified CLA).

|  | **Plasminogen activity** | **α2-plasmin inhibitor activity** | | **Factor XIII**  **activity** | | |
| --- | --- | --- | --- | --- | --- | --- |
| CLA parameters | | | | | | |
| maximal absorbance | r=0.63  95%CI:0.47-0.75  p<0.0001 | | r=0.52  95%CI:0.33-0.66  p<0.0001 | | r=0.42  95%CI:0.21-0.59  p=0.001 |  |
| time to maximal absorbance (min) | ns | | r=0.29  95%CI:0.07-0.48  p=0.01 | | ns |  |
| 10%CLT (min) | r=0.34  95%CI:0.13-0.53  p=0.002 | | r=0.23  95%CI:0.01-0.44  p=0.04 | | ns |  |
| 50%CLT (min) | r=0.29  95%CI:0.07-0.48  p=0.01 | | ns | | ns |  |
| 90%CLT (min) | r=0.29  95%CI:0.08-0.49  p=0.01 | | ns | | ns |  |
| CLA AUC (OD*min) | r=0.47  95%CI:0.27-0.63  p<0.001 | | r=0.26  95%CI:0.04-0.46  p=0.02 | | ns |  |
| Modified clot lysis assay (mCLA)* parameters | | | | | | |
| maximal absorbance | r=0.59  95%CI:0.43-0.72  p<0.0001 | | r=0.47  95%CI:0.27-0.63  p<0.001 | | r=0.39  95%CI:0.18-0.57  p=0.003 |  |
| time to maximal absorbance (min) | ns | | ns | | ns |  |
| 10%CLT (min) | ns | | ns | | ns |  |
| 50%CLT (min) | ns | | ns | | ns |  |
| 90%CLT (min) | r=0.25  95%CI:0.02-0.45  p=0.03 | | ns | | ns |  |
| CLA AUC (OD*min) | r=0.38  95%CI:0.17-0.56  p=0.005 | | ns | | ns |  |

*mCLA is performed in the presence of cell-free DNA and histones. 10%CLT, 10% clot-lysis time; 50%CLT, 50% clot-lysis time; 90%CLT, 90% clot-lysis time; 95%CI; 95% confidence interval, CLA, clot lysis assay; CLA AUC, clot lysis assay area under the curve
